# PDZK1 Prevents Neointima Formation via Suppression of Breakpoint Cluster Region Kinase in Vascular Smooth Muscle

**DOI:** 10.1371/journal.pone.0124494

**Published:** 2015-04-17

**Authors:** Wan Ru Lee, Anastasia Sacharidou, Erica Behling-Kelly, Sarah C. Oltmann, Weifei Zhu, Mohamed Ahmed, Robert D. Gerard, David Y. Hui, Jun-ichi Abe, Philip W. Shaul, Chieko Mineo

**Affiliations:** 1 Center for Pulmonary and Vascular Biology, Department of Pediatrics, University of Texas Southwestern Medical Center, Dallas, Texas, United States of America; 2 Department of Molecular Biology, University of Texas Southwestern Medical Center, Dallas, Texas, United States of America; 3 Department of Pathology, Metabolic Diseases Institute, University of Cincinnati College of Medicine, Cincinnati, Ohio, United States of America; 4 Department of Medicine and the Aab Cardiovascular Research Institute, University of Rochester School of Medicine and Dentistry, Rochester, New York, United States of America; University Hospital Würzburg, GERMANY

## Abstract

Scavenger receptor class B, type I (SR-BI) and its adaptor protein PDZK1 mediate responses to HDL cholesterol in endothelium. Whether the receptor-adaptor protein tandem serves functions in other vascular cell types is unknown. The current work determined the roles of SR-BI and PDZK1 in vascular smooth muscle (VSM). To evaluate possible VSM functions of SR-BI and PDZK1 in vivo, neointima formation was assessed 21 days post-ligation in the carotid arteries of wild-type, SR-BI^-/-^ or PDZK1^-/-^ mice. Whereas neointima development was negligible in wild-type and SR-BI^-/-^, there was marked neointima formation in PDZK1^-/-^ mice. PDZK1 expression was demonstrated in primary mouse VSM cells, and compared to wild-type cells, PDZK1^-/-^ VSM displayed exaggerated proliferation and migration in response to platelet derived growth factor (PDGF). Tandem affinity purification-mass spectrometry revealed that PDZK1 interacts with breakpoint cluster region kinase (Bcr), which contains a C-terminal PDZ binding sequence and is known to enhance responses to PDGF in VSM. PDZK1 interaction with Bcr in VSM was demonstrated by pull-down and by coimmunoprecipitation, and the augmented proliferative response to PDGF in PDZK1^-/-^ VSM was abrogated by Bcr depletion. Furthermore, compared with wild-type Bcr overexpression, the introduction of a Bcr mutant incapable of PDZK1 binding into VSM cells yielded an exaggerated proliferative response to PDGF. Thus, PDZK1 has novel SR-BI-independent function in VSM that affords protection from neointima formation, and this involves PDZK1 suppression of VSM cell proliferation via an inhibitory interaction with Bcr.

## Introduction

In endothelial cells, high density lipoprotein (HDL) cholesterol binding to scavenger receptor class B, type I (SR-BI) activates PI3 kinase and Akt kinase to stimulate endothelial NO synthase (eNOS) and endothelial cell migration[1,2]. Studies of carotid artery reendothelialization in SR-BI^+/+^ versus SR-BI^-/-^ mice indicate that the latter process is operative in vivo. The signaling capacity of SR-BI in endothelium requires its C-terminal PDZ interacting domain and the PDZ domain-containing adaptor protein PDZK1. SR-BI interaction with PDZK1 is also critical to SR-BI function in the liver, where the receptor promotes reverse cholesterol transport and thereby plays an important role in the regulation of global cholesterol homeostasis. Lack of interaction between PDZK1 and SR-BI in hepatocytes diminishes the abundance of SR-BI protein in the liver by 95%, leading to an increase in plasma total cholesterol carried in abnormally large HDL particles [3,4]. In contrast, in endothelial cells PDZK1 does not influence SR-BI protein stability and instead it is critical to the coupling of SR-BI to kinases and downstream cellular responses to HDL.

Whereas we have considerable knowledge of the vascular cell biology of SR-BI and PDZK1 in endothelium, the functions of the receptor and adaptor protein in vascular smooth muscle (VSM) are unknown. Dysregulation of VSM cell behavior plays a major role in neointima development, which participates in the pathogenesis of nonthrombotic vascular occlusion, restenosis, atherosclerosis and vein-graft failure[5]. These processes entail exaggerated VSM proliferation and migration prompted by inflammatory cytokines and growth factors such as tumor necrosis factor-α and platelet-derived growth factor (PDGF)[5–8]. In the current work, we sought to determine the roles of SR-BI and PDZK1 in VSM. This was first accomplished by evaluating the impact of SR-BI or PDZK1 deletion on neointima formation in response to carotid artery ligation in mice. Although the absence of SR-BI had no effect, PDZK1^-/-^ mice uniquely demonstrated exaggerated neointima development. This led to the discoveries that PDZK1 negatively regulates VSM cell growth and migration, and that this occurs via an inhibitory interaction of PDZK1 with breakpoint cluster region kinase (Bcr). Based on these findings, PDZK1 now warrants consideration as a modifier of neointima formation and restenosis.

## Materials and Methods

### Animal models

Experiments were performed in wild-type mice and in SR-BI^-/-^ or PDZK1^-/-^ mice on the same C57BL/6J and 129Sv/Ev mixed background[9,10]. The care and use of all study animals was approved by the Institutional Animal Care and Use Committee at UT Southwestern, and conducted in accordance with PHS Policy on the Humane Care and Use of Laboratory Animals.

### Neointima formation models

Neointima formation was evaluated following carotid artery ligation or femoral artery cuff placement in male mice (10–12 weeks of age), using methods modified from those previously described[11–13]. To study carotid artery neointima formation mice were anesthetized with Avertin, and the left common carotid artery was ligated just proximal to the carotid bifurcation. Mice were closely monitored during recovery for signs of stroke. Twenty-one days post-injury the mice were euthanized and perfused with 10% buffered formalin, and the carotid arteries were dissected from superior to the ligation site to the aortic arch, fixed in 10% buffered formalin for 48h, transferred to PBS and embedded in paraffin with the aortic arch oriented deep into the block. The tissue was sectioned until the ligature was visualized, and then the next 1.3 mm of tissue proximal to the ligature was removed by measured sectioning. Serial 4 μm sections were collected at this point and affixed to charged glass slides. To study neointima formation in the femoral artery, mice were anesthetized with Avertin and the left femoral artery was isolated from surrounding tissues. A polyethylene tube (2 mm long, PE-90, inner diameter 0.86 mm, outer diameter 1.27 mm, Becton-Dickinson) was placed around the artery. Thirty-five days post-surgery, mice were euthanized and perfused with 10% formalin, and the femoral arteries were dissected. Carotid and femoral artery sections were stained with hematoxylin and eosin and elastin was detected using Hart's staining, and carotid artery sections additionally underwent immunofluorescence analysis with anti-α-smooth muscle actin (α-SMA) monoclonal antibody to identify VSM cells (Sigma, clone 1A4) and immunohistochemical analysis with anti-F4/80 polyclonal antibody (Santa Cruz Biotechnology) to identify macrophages. In both models morphometric analysis was performed on four sections per animal using NIH IMAGE J software, and data were averaged for each animal. Medial area was calculated as the area encircled by external elastic lamina minus the area inside the internal elastic lamina, and intimal area was calculated as the area inside the internal elastic lamina minus the luminal area. The primary endpoint was the intima-to-media (IM) ratio.

### Primary VSM cell isolation and phenotyping

Aortic VSM cells were isolated from PDZK1^+/+^ or PDZK1^-/-^ littermates using previously-reported procedures with some modifications[14]. Briefly, thoracic aortas were dissected from the mice, adventitial tissue was removed, and the aortas were incubated in Hanks' solution containing 1 mg/ml collagenase and 0.5 mg/ml elastase for 30 min at 37°C. The resulting cell suspension was centrifuged at 150 × g for 5 min and resuspended in DMEM containing 10% fetal bovine serum. The identity of the isolated cells as VSM cells was confirmed by positive immunostaining with anti-α-SMA monoclonal antibody (Sigma, clone 1A4). Cells were maintained in DMEM containing 10% fetal bovine serum, 100 units/ml penicillin, and 0.1 mg/ml streptomycin, and experiments were done at passage 2–3.

VSM cell proliferation was assessed by previously described methods[15]. Briefly, cells were seeded in 96-well culture plates at 1x 10^4^ cells per well and cultured in DMEM containing 0.2% fetal bovine serum for 48h. The cells were incubated with 0–20 ng/ml PDGF-BB (Invitrogen) for 18h at 37°C followed by incubation with [^3^H] thymidine (0.2 μCi/well, 28 nM, PerkinElmer) for 6h, washed with phosphate-buffered saline (PBS) and lysed with 0.25 M sodium hydroxide, and radioactivity in cell lysates was quantified by liquid scintillation counting.

VSM cell migration was evaluated in a Transwell apparatus using a membrane filter with pore size of 8.0 μm[15]. Following plating of 2 X 10^4^ cells/well, cells were treated with 0–30 ng/ml PDGF-BB for 4h. Nonmigrating cells on the upper surface were scraped gently and removed by PBS wash and wiping with a cotton-tipped applicator. The filters were fixed in ice-cold methanol and stained with DAPI, and the number of cells per high-power field which had migrated into the membrane was counted microscopically (NIKON Eclipse TE2000-E, x20 magnification, 5 fields per chamber). All cell culture findings were replicated in 3 independent experiments.

### Quantitative RT PCR

Total RNA was isolated using TRIzol Reagent (Life Technologies, Carlsbad, CA) according to the manufacturer’s protocol and stored at −80°C. Reverse transcription was performed using QuantiTect Reverse Transcription kit (Qiagen, Valencia, CA). cDNA for RT PCR was generated using gene specific primers for mouse PDZK1 and the housekeeping gene cyclophilin as follows: PDZK1 sense, 5’ AGGATCAATGGTGTCTTTGTCG 3’, PDZK1 anti-sense, 5’ TCCAGCTCTTTCAAATCCACC 3’, Cyclophilin sense, 5’ TGGAGAGCACCAAGACAGACA 3’, Cyclophilin anti-sense, 5’ TGCCGGAGTCGACAATGAT 3’. PCR was carried out using SYBR Green PCR Master Mix from PE Applied Biosystems (Warrington, UK) on an Applied Biosystems Prism 7900HT Sequence Detection System (PE Applied Biosystems).

### Tandem affinity purification (TAP)

Adenoviral constructs were engineered containing a TAP tag, which consisted of two protein G sequences and a streptavidin-binding peptide separated by a TEV protease cleavage site[16,17], linked to either full-length PDZK1 (Tag-PDZK1) or TAP tag alone. HEK293 cells (American Tissue Culture Collection) were infected with the adenoviruses and 60h later cell lysates were obtained and subjected sequentially to IgG-Sepharose bead binding, TEV protease cleavage, and streptavidin-Sepharose bead binding. The final eluents containing overexpressed Tag-PDZK1 or Tag alone and associated proteins were separated by SDS-PAGE, the obtained proteins were proteolytically digested, and subjected to mass spectrometry (MS) analysis[18]. The resulting MS data were analyzed using Mascot algorithm software to obtain the identity of the proteins[19].

### Bcr-PDZK1 Interaction

To assess the interaction between PDZK1 and Bcr in VSM cells, pull-down experiment was performed. Primary mouse VSM cells were infected with adenovirus expressing Tag alone or Tag-PDZK1, and 60h later the cells were harvested in lysis buffer (50 mM HEPES-KOH/pH7.5, 10% glycerol, 100mM KCl, 2mM EDTA, 0.1% NP-40, 10mM NaF, 0.25 mM Na_3_VO_4_, 50mM β-glycerolphosphate, 2mM DTT, Protease inhibitors cocktail (Sigma-Aldrich)). IgG-Sepharose beads were added to the cell lysate and incubated for 4h at 4°C, the beads were washed three times with TEV buffer (50 mM Tris-HCl/pH8.0, 0.5mM EDTA, 1mM DTT) and the bound proteins were eluted by boiling for 5 min in SDS sample buffer. Eluted proteins were separated by SDS-PAGE, and immunoblot analysis was performed using anti-Bcr polyclonal antibody (Santa Cruz Biotechnology).

To evaluate interactions between endogenous PDK1 and Bcr in VSM cells, coimmunoprecipitation experiments were performed. Primary mouse VSM cells were lysed with 1% TX-100 buffer (150mM NaCl, 100mM Tris-HCL pH8, 1% TX-100, 1mM MgCl_2_, 1mM CaCl_2_) and incubated with anti-Bcr polyclonal antibody (1.5 μg/ml) or anti-PDKZ1 monoclonal antibody (1.5 μg/ml, Santa Cruz Biotechnology) in the presence of Protein A/G PLUS agarose beads (Santa Cruz, cat#sc-2003) at 4°C for 2h. Immunoprecipitates were washed 3 times with 0.1% TX-100 buffer (150mM NaCl, 100mM Tris-HCL pH8, 0.1% TX-100, 1mM MgCl_2_, 1mM CaCl_2_) and proteins were eluted by boiling for 5 min in SDS sample buffer. Eluted proteins were separated by SDS-PAGE, and immunoblot analysis was performed using anti-Bcr antibody and anti-PDZK1 antibody.

### Bcr manipulation in VSM cells

To delete Bcr from VSM cells, double stranded RNAs with sequences 5’-GAAUGGAAUUGAAGUGAAAUU-3’ and 5’- UUUCACUUCAAUUCCAUUCUU-3’ were designed to target the open reading frames of mouse Bcr (NM_001081412). Non-targeting siRNA was used as a negative control (ON-TARGETplus Non-targeting siRNA, Dharmacon, D-001810-02-20). VSM cells were transfected with 30 nM dsRNA using RNAiFect (Qiagen) following the manufacturer's instruction, and 48h later the cells were placed in DMEM containing 0.2% fetal bovine serum for 48h for proliferation assays.

The Bcr-V1271A mutant, which has reduced interaction with PDZK1[20] was generated by site-directed mutagenesis (Clontech). Wild-type and mutant Bcr cDNA were subcloned into pACCMVpLpA(-)loxP-SSP shuttle vector (http://www2.med.umich.edu/medschool/vcore/PlasmidList.cfm), and recombinant adenoviruses were constructed by in vitro cre/loxP-mediated recombination[21,22]. Constructs were propagated in 911 cells, medium was collected, and plaque assays were performed to determine virus titers. Adenovirus derived from empty shuttle vector (AdCMVpLpA-loxP) was used as a negative control. VSM cells were incubated with adenovirus for 64h, and the abundance of Bcr protein was determined by immunoblot analysis using anti-Bcr polyclonal antibody (Santa Cruz Biotechnology) or anti-GAPDH monoclonal antibody (Ambion) to evaluate protein loading.

### Ethics Statement

The care and use of all study animals was approved by the Institutional Animal Care and Use Committee at UT Southwestern (Animal Protocol Number 2010–0046), and conducted in accordance with PHS Policy on the Humane Care and Use of Laboratory Animals. For studies evaluating neointima formation, mice were anesthetized by IP injection of Tribromoethanol (250 mg/kg), and mice were euthanized by 2% isoflurane inhalation followed by cervical dislocation.

### Statistical analysis

Comparisons between two groups were performed by Student’s t tests. Differences between multiple groups were evaluated by one-way analysis of variance (ANOVA) with Tukey's post-hoc testing. When indicated, nonparametric ANOVA (Kruskal-Wallace) and post-hoc Dunn testing was performed. Significance was defined as p<0.05.

## Results

### SR-BI, PDZK1 and neointima formation

**Fig 1:**
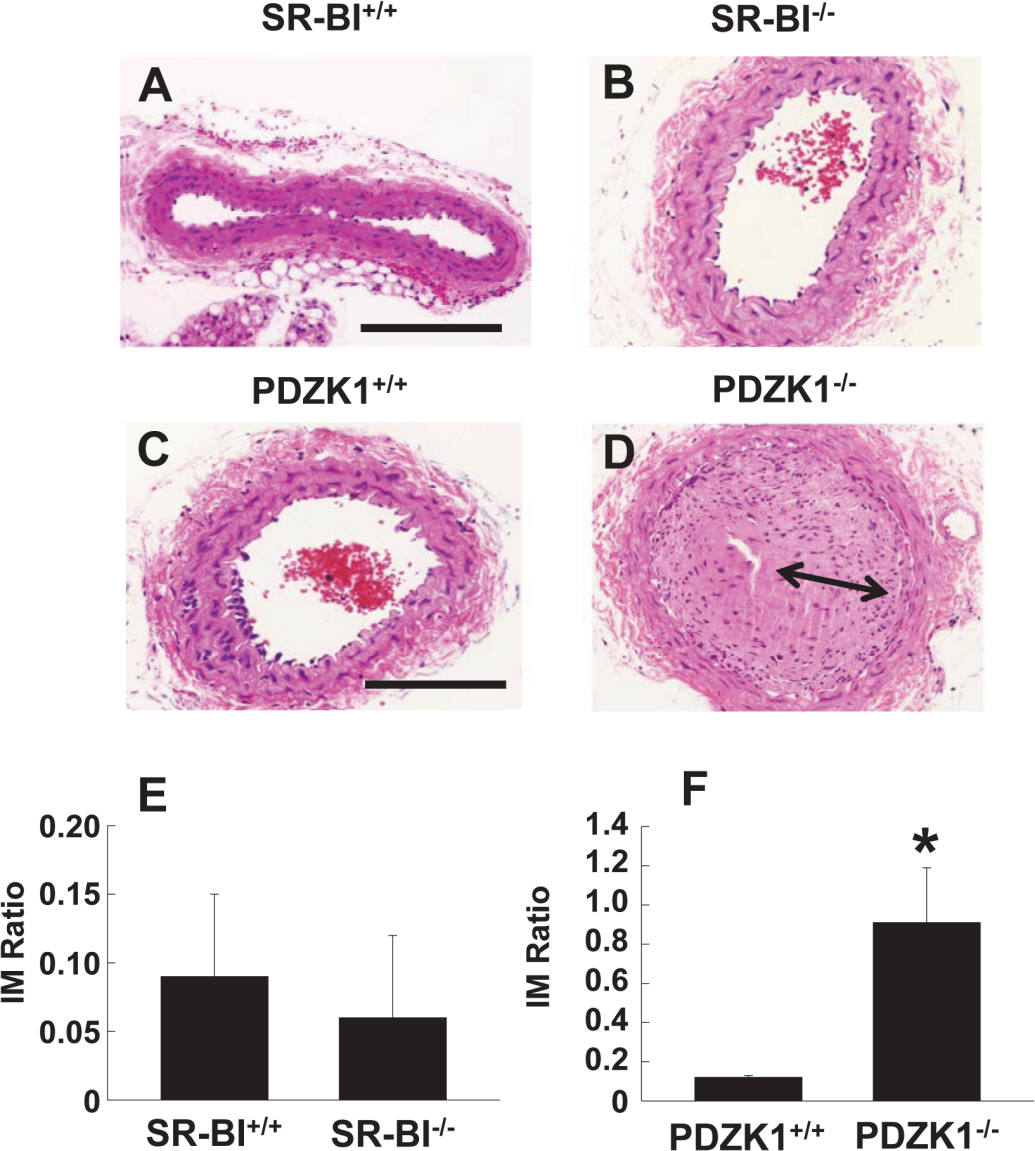
PDZK1 deletion causes exaggerated carotid artery neointima formation. A, B. Male SR-BI^+/+^ and SR-BI^-/-^ mice (10–12 weeks of age) underwent unilateral left carotid artery ligation for the evaluation of neointima formation. Arteries were harvested 21d later, and representative images of sections from SR-BI^+/+^ and SR-BI^-/-^ mice stained with hematoxylin and eosin are shown. The scale bar indicates 100 μm. C, D. Neointima formation was also evaluated in male PDZK1^+/+^ and PDZK1^-/-^ mice. Neointima is indicated by arrow. E. Summary data for intima-to-media (IM) ratio for SR-BI^+/+^ versus SR-BI^-/-^. F. Summary data for IM ratio for PDZK1^+/+^ versus PDZK1^-/-^. In E and F, values are mean±SEM, n = 7–9, *p<0.05 vs. PDZK1^+/+^.

To determine how SR-BI impacts neointima formation, neointima development following carotid artery ligation was evaluated in SR-BI^+/+^ and SR-BI^-/-^ mice. The left common carotid arteries of male 10–12 week old mice were ligated, and twenty-one days post-ligation the arteries were harvested and vascular remodeling was assessed. In wild-type SR-BI^+/+^ mice, the ligation induced minimal neointima formation (Fig 1A and 1E). There was also negligible neointima development in SR-BI^-/-^ mice (Fig 1B and 1E). Carotid artery ligations were also performed in PDZK1^+/+^ versus PDZK1^-/-^ mice, and there was minimal neointima development in PDZK1^+/+^ (Fig 1C). In contrast, there was marked neointima formation in PDZK1^-/-^ mice (Fig 1D). Summary data indicated that the intima-media (IM) ratio was 7.6-fold greater in PDZK1^-/-^ versus PDZK1^+/+^ mice (Fig 1F). To confirm the finding of exaggerated neointima formation in PDZK1^-/-^ carotid arteries, neointima development following femoral artery cuff placement was also evaluated (S1 Fig). Whereas minimal neointima development was observed in PDZK1^+/+^ mice (S1 Fig Panel A), there was marked neointima formation in PDZK1^-/-^ mice (S1 Fig Panel B). Summary data indicated that the IM ratio was 7.2-fold greater in PDZK1^-/-^ compared to PDZK1^+/+^ mice (S1 Fig Panel C), mirroring the observations made in the carotid artery.

**Fig 2:**
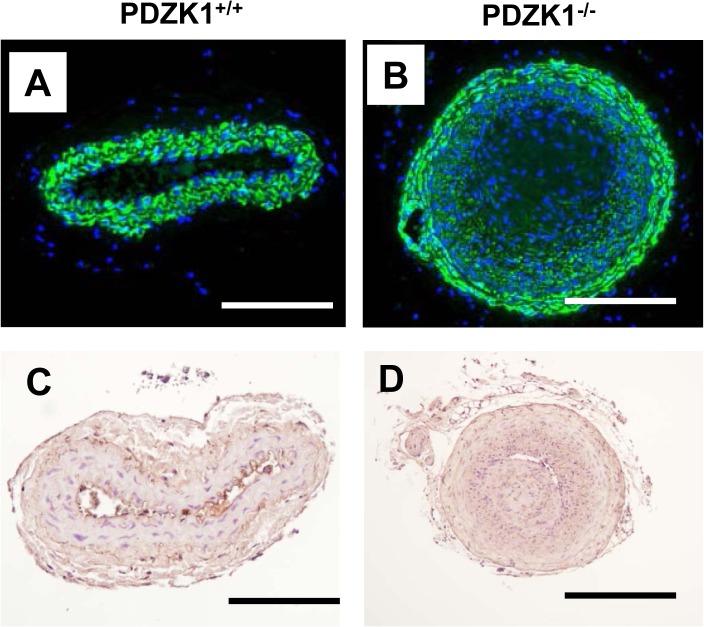
VSM cells populate the neointima that develops in PDZK1-/- mice. Male wild-type PDZK1^+/+^ (A, C) and PDZK1^-/-^ mice (B, D) underwent unilateral left carotid artery ligation for the evaluation of neointima formation. Arteries were harvested 21d later, and immunofluorescent staining was performed with anti-αSMA monoclonal antibody to detect smooth muscle cells (A and B, green fluorescent staining for αSMA and blue for DAPI), and immunohistochemistry was done with anti-F4/80-polyclonal antibody to detect macrophages (C and D). Positive F4/80 signal is seen in rare luminal macrophages in C. The scale bar indicates 100 μm.

The composition of the neointima formed in the PDZK1^-/-^ mice was then assessed. Immunofluorescence staining for the smooth muscle cell marker α-smooth muscle actin (αSMA) showed abundant VSM cells in the neointima (Fig 2A and 2B). More intense staining for αSMA was observed towards the periphery of the neointima area compared to the inner core of the lesion, but the central region did display positive signal. Immunohistochemical staining for F4/80 protein revealed minimal macrophage infiltration in the lesions (Fig 2C and 2D). Thus, the global loss of PDZK1 resulted in exaggerated formation of VSM cell-populated neointima, differing markedly from the negligible impact of SR-BI deletion. These findings contrast with the parallel attenuation of endothelial cell repair observed in SR-BI^-/-^ and PDZK1^-/-^ mice, and the partnership between the receptor and adaptor protein that is required for HDL modulation of eNOS activity [10,23]. These observations suggest that PDZK1 has SR-BI-independent function in VSM that tempers VSM cell proliferation and/or migration.

### PDZK1 modulation of VSM cell proliferation and migration

**Fig 3:**
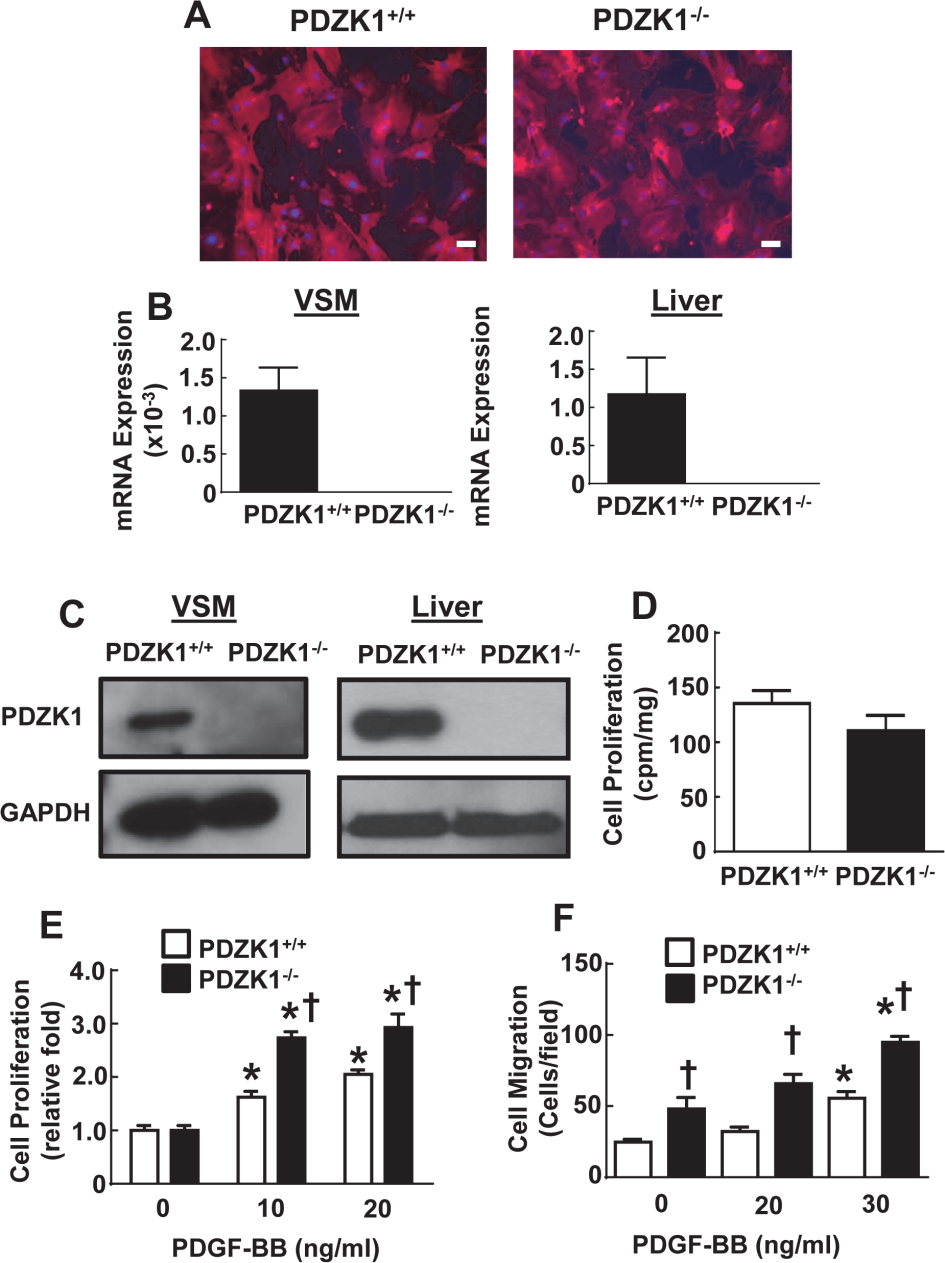
VSM cells from PDZK1-/- mice display enhanced PDGF-stimulated cell proliferation and migration. A. Aortic VSM cells were isolated from PDZK1^+/+^ or PDZK1^-/-^ mice and immmunfluorescent staining was performed with anti-α-SMA monoclonal antibody (red). The scale bar indicates 100 μm. B. PDZK1 mRNA was quantified in VSM cells and liver from PDZK1^+/+^ and PDZK1^-/-^ mice. Results are expressed relative to cyclophilin. C. PDZK1 protein expression was evaluated by immunoblotting using anti-PDZK1 polyclonal antibody. Equal protein loading was confirmed by immunoblotting using anti-GAPDH antibody. D. Cell growth under quiescent conditions was assessed by quantification of ^3^H-thymidine incorporation. E.Cell proliferation was evaluated in VSM incubated in the presence of 0–20 ng/ml PDGF-BB for 24h. F. Cell migration was assessed in VSM incubated in the presence of 0–30 ng/ml PDGF-BB for 4h. In B and D-F, values are mean±SEM, n = 3, *p<0.05 vs. no PDGF, †p<0.05 vs. PDZK1^+/+^.

To directly determine if PDZK1 influences the proliferation or migration of VSM cells, primary VSM cells were isolated from the aortas of wild-type PDZK1^+/+^ and PDZK1^-/-^ mice by enzymatic dispersion [24]. The early passage VSM cells from the two genotype groups were indistinguishable, displaying a typical spindle shape and filamentous αSMA bundles spanning the length of the cells, and more than 90% of cells displayed positive immunohistochemical staining for αSMA (Fig 3A). PDZK1 expression in VSM cells was then evaluated by RT-PCR and immunoblotting, using liver as a positive control tissue (Fig 3B and 3C); PDZK1 transcript and protein were detectable in PDZK1^+/+^ VSM and they were predictably absent in PDZK1^-/-^ cells. To assess the relative expression of PDZK1 in VSM, parallel quantification of the transcript was done in liver where PDZK1 expression is substantial [25]. The abundance of PDZK1 mRNA in VSM was less than 0.2% of that found in the liver.

Cell proliferation were then compared in PDZK1^+/+^ versus PDZK1^-/-^ VSM cells under quiescent conditions by measurements of ^3^H-thymidine incorporation, and they were found to be similar (Fig 3 D). However, PDGF-stimulated proliferation was augmented in PDZK1^-/-^ VSM cells (Fig 3 E). PDGF-induced cell migration was evaluated using a Transwell apparatus, and it too was greater in PDZK1^-/-^ VSM cells (Fig 3 F). Thus, the absence of PDZK1 enhances both VSM cell proliferation and migration responses to PDGF, indicating that PDZK1 is a negative modulator of these critical VSM functions. These findings provide a plausible explanation for the exaggerated neointima formation observed in PDZK1^-/-^ mice.

### Bcr interaction with PDZK1 in VSM cells

PDZK1 is an adaptor protein containing four PDZ domains mediating its interaction with other proteins, and PDZK1 has no other known functional domains [25,26]. Recognizing that it is an adaptor protein, to elucidate the molecular basis for PDZK1 regulation of VSM cells, tandem affinity purification (TAP) was performed to identify proteins that interact with PDZK1. This two-step purification strategy enables the isolation of protein complexes under close to physiologic conditions [16,17]. HEK293 cells were infected with adenoviruses expressing either a TAP Tag alone or a fusion protein comprised of the TAP tag and PDZK1 (Tag-PDZK1). The two-step purification procedure was performed 48h later, and the proteins obtained were subjected to SDS-PAGE. Proteins captured by the fusion protein with PDZK1 versus tag alone were purified and peptide sequences were obtained by mass spectrometry. The sequences of multiple peptides identified Bcr kinase as a PDZK1-interacting protein (S1 Table).

**Fig 4:**
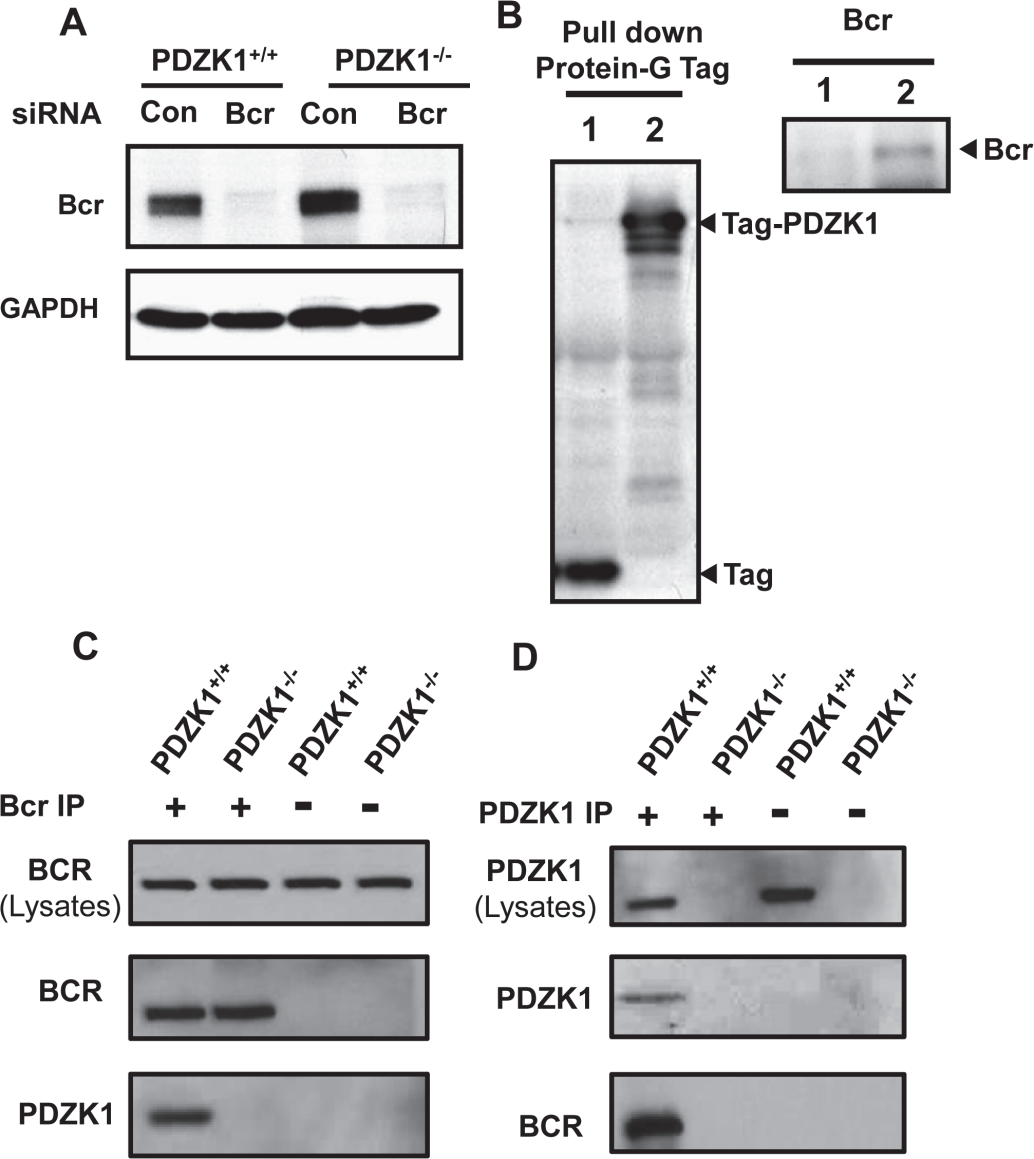
Bcr is expressed in VSM cells and interacts with PDZK1. A. PDZK1^+/+^ or PDZK1^-/-^ VSM cells were transfected with control dsRNA (Con) or dsRNA targeting Bcr, and Bcr expression was evaluated by immunoblot analysis 24h later. B. Wild-type VSM cells were infected with adenovirus expressing protein G sequence-containing Tag alone (lane 1) or Tag-PDZK1 (lane 2), and 60h later the cells were lysed and Tag or Tag-PDZK1 were precipitated using IgG sepharose. Tag and Tag-PDZK1 were detected by HRP-linked secondary antibody binding to the Protein G sequence in the Tag (left panel). The pull-down of Bcr was detected by immunoblotting of samples from Tag-PDZK1 infected cells (right panel). C. Endogenous Bcr was immunoprecipitated from PDZK1^+/+^ or PDZK^-/-^ VSM cells by anti-Bcr antibody, and the presence of Bcr and PDZK1 in the immunoprecipitates was detected by immunoblotting. D. Endogenous PDZK1 was immunoprecipitated by anti-PDZK1 antibody, and Bcr and PDZK1 in the immunoprecipitates was detected by immunoblotting.

Bcr was originally identified as the breakpoint of the Philadelphia chromosome translocation associated with chronic myelogenous leukemia [27,28]. It is a 160 kDa protein that contains a number of putative functional domains, including a C-terminal sequence (STEV) that is a ligand for PDZ domains [27,29,30]. Previous studies demonstrated that Bcr is expressed in VSM cells, and that its function enhances VSM proliferation [31,32]. The expression of Bcr in primary VSM cells was confirmed by immunoblot analysis of cells transfected with control siRNA versus siRNA targeting the kinase (Fig 4A), and there was no difference in the levels of Bcr expression in PDZK1^+/+^ versus PDZK1^-/-^ VSM. The interaction of Bcr with PDZK1 in VSM cells was evaluated by pull-down using protein G in the TAP-Tag (Fig 4B). VSM cells from wild-type mice were infected with adenovirus encoding either the TAP-Tag alone, which contains two protein G sequences, or the Tag-PDZK1. Tag or Tag-PDZK1 proteins were captured using IgG Sepharose beads, and Bcr co-precipitated with PDZK1 was detected by immunoblot analysis. Interaction between endogenous PDZK1 and Bcr was evaluated in VSM cells from PDKZ1^+/+^ or PDZK1^-/-^ mice in coimmunoprecipitation experiments. In PDZK1^+/+^ cells, PDZK1 was coimmunoprecipitated with Bcr (Fig 4C), and in a reciprocal manner, Bcr was coimmunoprecipitated with PDZK1 (Fig 4D). These results indicate that Bcr is a PDZK1 interacting protein in VSM cells.

### Role of Bcr in PDZK1 action in VSM cells

**Fig 5:**
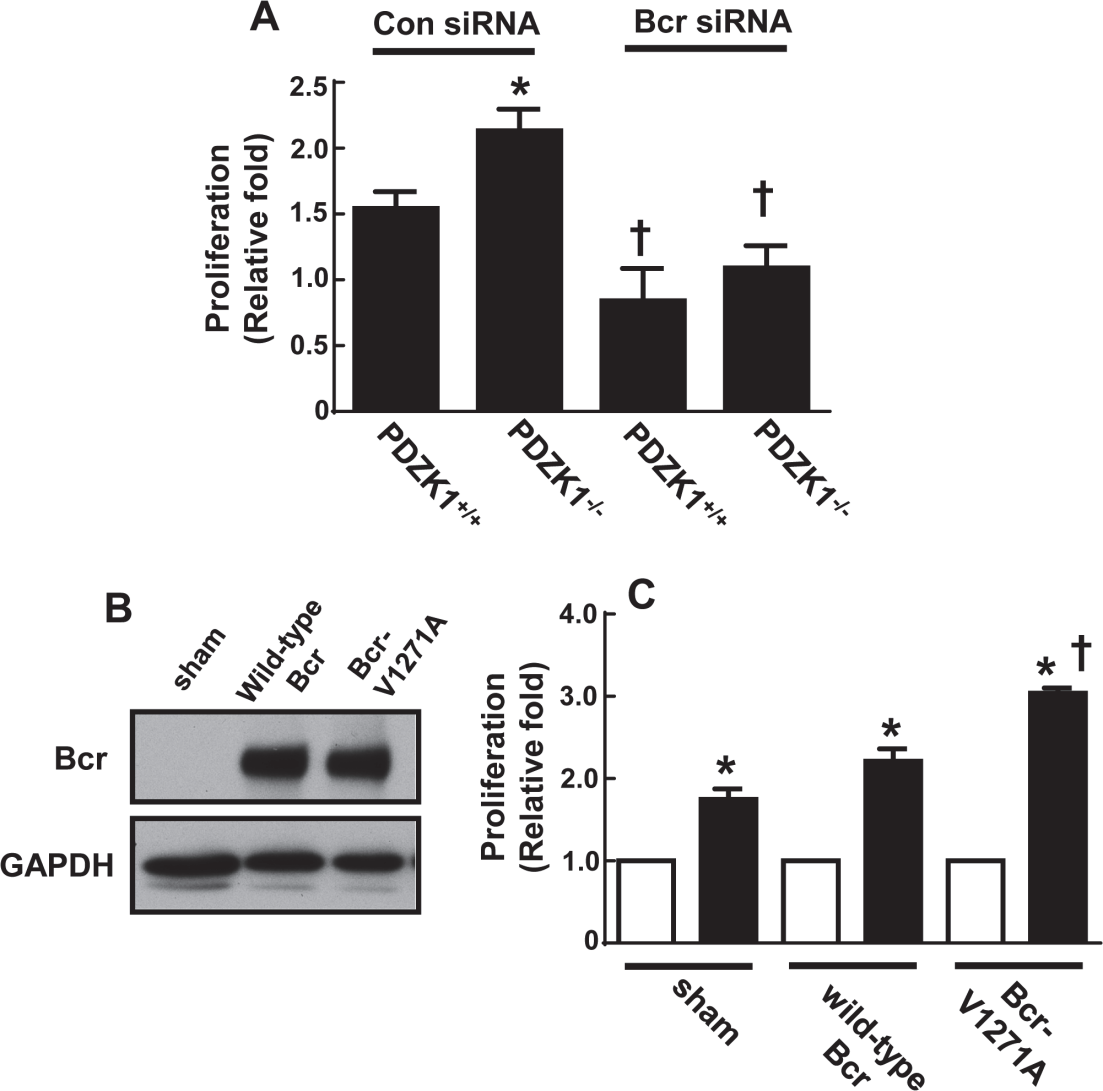
Bcr interaction with PDZK1 blunts VSM cell proliferation. A. VSM cells from PDZK1+/+ or PDZK1-/- mice were transfected with control dsRNA (Con siRNA) or dsRNA targeting Bcr (Bcr siRNA), and cell proliferation in response to PDGF-BB (10 ng/ml) was assessed by quantification of 3H-thymidine incorporation. Effective knockdown of Bcr is shown in Fig 4A. PDGF-BB-stimulated proliferation is expressed relative to proliferation in control, PBS-treated cells. Values are mean±SEM, n = 8, *p<0.05 vs. PDZK1^+/+^, †p<0.05 vs. Con siRNA. B. VSM cells were infected with adenovirus expressing vector alone (sham), wild-type Bcr or Bcr-V1271A, and relative Bcr expression was evaluated by immunoblot analysis. C. In cells described in B, proliferation was assessed in control PBS-treated cells (open bar) and in response to PDGF-BB (10 ng/ml, closed bar). PDGF-BB-stimulated proliferation is expressed relative to proliferation in control-treated cells. Values are mean±SEM, n = 3, *p<0.05 vs. no PDGF, †p<0.05 vs. wild-type Bcr.

It was next determined whether Bcr is required for the exaggerated proliferation observed in response to PDGF in PDZK1^-/-^ VSM cells using siRNA knockdown of the kinase. In siRNA control-transfected cells, PDZK1^-/-^ cells showed exaggerated proliferation in response to PDGF (10 ng/ml) versus PDZK1^+/+^ cells (Fig 5A), confirming the results shown in Fig 3E. However, PDGF-stimulated proliferation was comparable in PDZK1^+/+^ versus PDZK1^-/-^ cells depleted of Bcr. To determine how interaction with PDZK1 affects Bcr function in VSM, wild-type VSM cells were transfected with sham vector, cDNA encoding wild-type Bcr, or cDNA encoding a mutant form of Bcr with a disrupted C-terminal PDZ binding motif (Bcr-V1271A) that diminishes the interaction between Bcr and PDZK1 [20](Fig 5B). Compared to wild-type Bcr overexpression, the introduction of the Bcr-V1271A mutant yielded an exaggerated proliferation response to PDGF (Fig 5C). These findings indicate that Bcr protein is required for the exaggerated proliferation observed in PDZK1^-/-^ VSM cells, and that the disruption of Bcr-PDZK1 interaction enhances VSM cell proliferation with PDGF. As such, PDZK1 tempers VSM cell proliferation via an inhibitory interaction with Bcr, revealing a mechanism whereby PDZK1 may afford protection from neointima formation.

## Discussion

In addition to its participation in global cholesterol homeostasis and reverse cholesterol transport from peripheral tissues and cells to the liver, HDL cholesterol has direct action on vascular endothelium. Via signaling prompted by HDL-induced cholesterol movement, SR-BI and PDZK1 activate kinases and thereby stimulate eNOS and endothelial cell migration[9,33–35]. Whereas we have substantial understanding of the biology of this HDL receptor-adaptor protein pair in endothelium, their potential roles in other vascular cells are unknown. Using a model of neointima formation in the carotid artery, we discovered that although SR-BI deletion has no impact on neointima development, PDZK1 deficiency results in exaggerated neointima formation. The impact of PDZK1 on neointima development was confirmed using a femoral artery cuff model of the disorder. Paralleling the observation in vivo, PDZK1^-/-^ VSM cells have enhanced proliferation and migration responses to PDGF. Thus, we have revealed that PDZK1 has unique, SR-BI-independent function in VSM involved in the modulation of neointima formation.

A role for PDZK1 in atheroprotection was previously revealed in studies in which western diet-fed apoE^-/-^;PDZK1^-/-^ mice displayed greater atherosclerotic plaque formation than apoE^-/-^ mice[36,37]. The increase in atherosclerosis in the absence of PDZK1 has been attributed to the loss of PDZK1 preservation of SR-BI protein stability in the liver and a resulting marked increase in plasma cholesterol carried in abnormally large HDL particles enriched in unesterified cholesterol, which also occurs in SR-BI^-/-^ mice [4,38]. In apoE^-/-^;PDZK1^-/-^ mice, receipt of a Paigen diet results in further exaggeration of atherosclerosis with macrophage-rich lesions and occlusive coronary artery disease in association with extreme hypercholesterolemia. The phenotypes in this model have also been primarily attributed to alterations in SR-BI function resulting from the loss of PDZK1[37]. The present work is the first to identify a function of PDZK1 that impacts cardiovascular health independent of its modulation of SR-BI abundance or action.

Recognizing the capacity of PDZ domains to scaffold protein networks, an unbiased approach was taken to identify PDZK1 binding proteins. Using tandem affinity purification and mass spectrometry, we identified 22 proteins that interact with PDZK1 in HEK293 cells. The protein with the highest Mascot score was identified to be Bcr, which was originally defined as the breakpoint of the Philadelphia chromosome translocation associated with chronic myelogenous leukemia [27,28]. In addition to its C-terminal STEV sequence that is a ligand for PDZ domain binding, Bcr contains a serine/threonine protein kinase domain, a guanine nucleotide exchange factor domain and a GTP-ase activating protein domain, and the two latter domains regulate the activity of Rho-type GTPases[29,30]. There is also an oligomerization domain at its N-terminus, a plekstrin homology domain, and a Src-homology 2-binding domain[27]. Bcr-PDZK1 interaction was previously reported in human lung-derived epithelial cells, and pull-down studies determined that the interaction involves the first PDZ domain of PDZK1[20]. However, the mechanistic relevance of PDZK1-Bcr interaction has not been previously interrogated in any paradigm[20]. In VSM, Bcr promotes responses to PDGF and Ang II that entail the activation of ERK1/2 and PPARγ and lead to increases in DNA synthesis and proinflammatory processes[31,32]. Bcr expression is upregulated in the neointima following balloon arterial injury in rats [32], and compared to wild-type mice, Bcr null mice display less neointima formation and reduced VSM cell proliferation following carotid artery ligation[31]. In the present work we demonstrate that Bcr deletion tempers the enhancement in PDGF-induced cell proliferation that occurs with PDZK1 loss from VSM cells, and that the interaction between Bcr and PDZK1 attenuates the pro-proliferative actions of the kinase in VSM. This mechanism provides a plausible explanation for how PDZK1 tempers neointima formation.

Having identified a novel function for PDZK1 in VSM as well as its basis, how PDZK1 expression in VSM may be modulated becomes a consideration. Information regarding the regulation of PDZK1 abundance in vivo is limited to studies of the adaptor protein in the liver. The administration of glucagon increases hepatic PDZK1 in rats[39], and fibrates dramatically suppress liver PDZK1 in mice[40]. In cell culture, it has been demonstrated that both peroxisome proliferator-activated receptor α (PPARα) agonists and estradiol induce PDZK1 protein expression in a human hepatoma cell line and in an ovarian cancer cell line, respectively[41] [42], and consensus sequences for a peroxisome proliferator responsive element and an estrogen responsive element have been identified in the human PDZK1 promoter[41]. Regarding the potential impact of pathologic conditions on PDZK1, it has been demonstrated that a proatherogenic diet reduces hepatic PDZK1 protein abundance[43]. It is unknown whether such a diet attenuates PDZK1 expression in VSM, thereby potentially contributing to aberrant VSM proliferative and migratory responses in the setting of hypercholesterolemia.

Uniquely impacting cardiovascular health in an SR-BI-independent manner, PDZK1 affords protection from neointima formation, and this is related to the suppression of VSM cell proliferation via an inhibitory interaction of PDZK1 with Bcr. Independent of plasma lipoproteins and SR-BI, PDZK1 now warrants consideration as a modifier of neointima formation and restenosis. In addition, these processes can potentially be harnessed to regulate VSM cell growth or migration.

## Supporting Information

S1 Fig PDZK1 deletion causes exaggerated femoral artery neointima formation. A, B. Male PDZK1^+/+^ and PDZK1^-/-^ mice (10–12 weeks of age) underwent unilateral left femoral artery cuff placement for the evaluation of neointima formation. Arteries were harvested 35d later, and representative images of sections from PDZK1^+/+^ and PDZK1^-/-^ mice stained with hematoxylin and eosin are shown. The scale bar indicates 100 μm. C. Summary data for IM ratio for PDZK1^+/+^ versus PDZK1^-/-^. Values are mean±SEM, n = 4, *p<0.05 vs. PDZK1^+/+^.
(PPTX)

S1 Table PDZK1 interacting proteins detected by tandem affinity purification. HEK293 cells were infected with adenoviral constructs encoding TAP Tag alone or TAP-PDZK1. Sixty hours later cell lysates were obtained and subjected to IgG-Sepharose bead and streptavidin-Sepharose bead binding. The final eluents containing Tag alone or Tag-PDZK1 and associated proteins were separated by SDS-PAGE and proteins uniquely obtained with PDZK1 precipitation were analyzed by mass spectrometry. Proteins with Mascot score >150 are listed. Peptides representing PDZK1 itself or the Tag are not included.
(PPTX)
