# Supplementary figures and images for: PDZK1 Prevents Neointima Formation via Suppression of Breakpoint Cluster Region Kinase in Vascular Smooth Muscle
Source: PLoS One. 2015 Apr 17;10(4):e0124494. doi: 10.1371/journal.pone.0124494 (PMC4401672; doi:10.1371/journal.pone.0124494)

## Slide 1
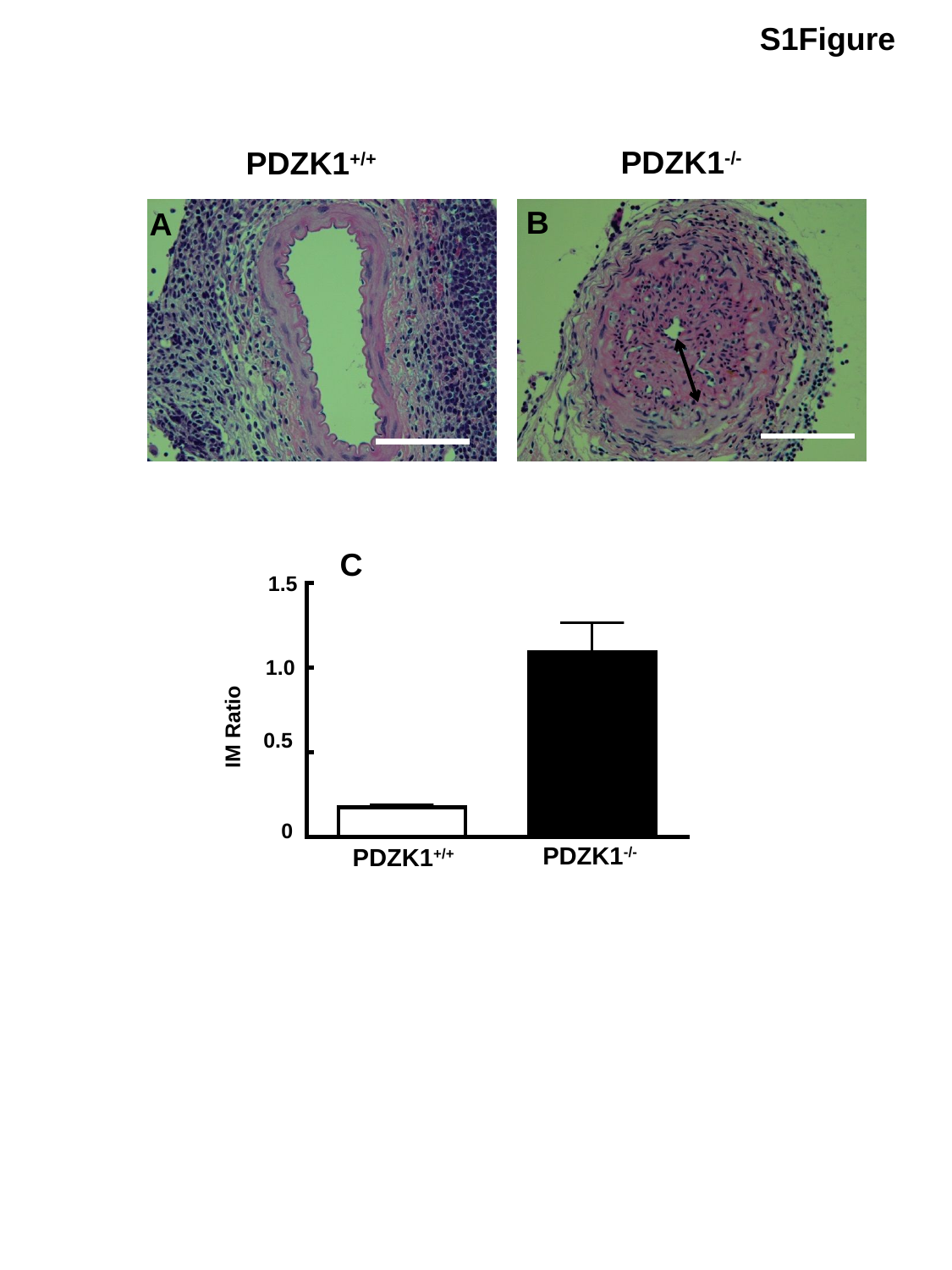

S1Figure
PDZK1-/-
PDZK1+/+
B
A
C
1.5
1.0
IM Ratio
0.5
0
PDZK1-/-
PDZK1+/+

Supplement: S1 Fig — A, B. Male PDZK1+/+ and PDZK1-/- mice (10–12 weeks of age) underwent unilateral left femoral artery cuff placement for the evaluation of neointima formation. Arteries were harvested 35d later, and representative images of sections from PDZK1+/+ and PDZK1-/- mice stained with hematoxylin and eosin are shown. The scale bar indicates 100 μm. C. Summary data for IM ratio for PDZK1+/+ versus PDZK1-/-. Values are mean±SEM, n = 4, *p<0.05 vs. PDZK1+/+. (PPTX) [file pone.0124494.s001.pptx]
