# Supplementary material for: PDZK1 Prevents Neointima Formation via Suppression of Breakpoint Cluster Region Kinase in Vascular Smooth Muscle
Source: PLoS One. 2015 Apr 17;10(4):e0124494. doi: 10.1371/journal.pone.0124494 (PMC4401672; doi:10.1371/journal.pone.0124494)
Supplement: S1 Table — HEK293 cells were infected with adenoviral constructs encoding TAP Tag alone or TAP-PDZK1. Sixty hours later cell lysates were obtained and subjected to IgG-Sepharose bead and streptavidin-Sepharose bead binding. The final eluents containing Tag alone or Tag-PDZK1 and associated proteins were separated by SDS-PAGE and proteins uniquely obtained with PDZK1 precipitation were analyzed by mass spectrometry. Proteins with Mascot score >150 are listed. Peptides representing PDZK1 itself or the Tag are not included. (PPTX) [file pone.0124494.s002.pptx]

## Slide 1
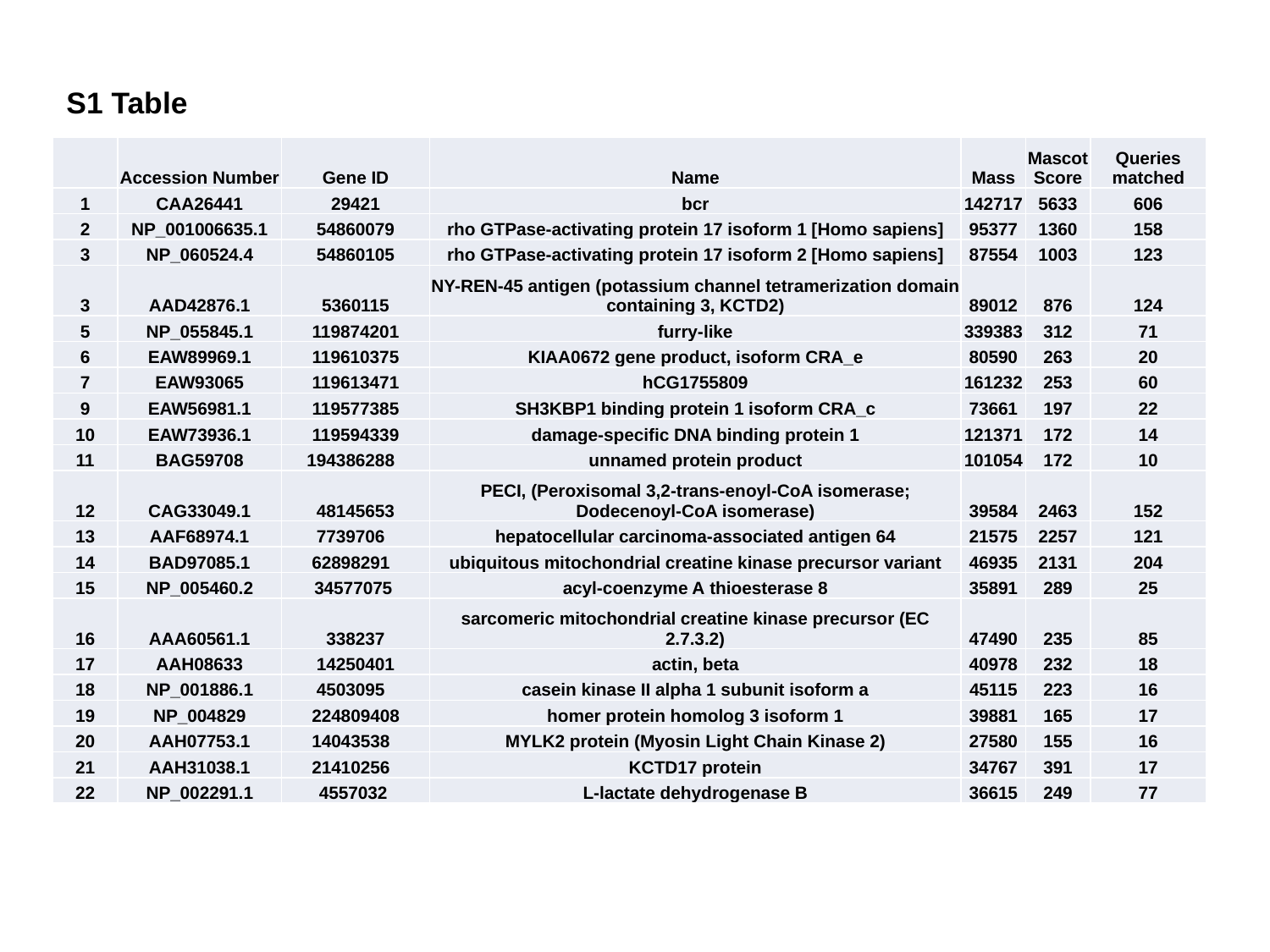

S1 Table
| | Accession Number | Gene ID | Name | Mass | MascotScore | Queries matched |
| --- | --- | --- | --- | --- | --- | --- |
| 1 | CAA26441 | 29421 | bcr | 142717 | 5633 | 606 |
| 2 | NP\_001006635.1 | 54860079 | rho GTPase-activating protein 17 isoform 1 [Homo sapiens] | 95377 | 1360 | 158 |
| 3 | NP\_060524.4 | 54860105 | rho GTPase-activating protein 17 isoform 2 [Homo sapiens] | 87554 | 1003 | 123 |
| 3 | AAD42876.1 | 5360115 | NY-REN-45 antigen (potassium channel tetramerization domain containing 3, KCTD2) | 89012 | 876 | 124 |
| 5 | NP\_055845.1 | 119874201 | furry-like | 339383 | 312 | 71 |
| 6 | EAW89969.1 | 119610375 | KIAA0672 gene product, isoform CRA\_e | 80590 | 263 | 20 |
| 7 | EAW93065 | 119613471 | hCG1755809 | 161232 | 253 | 60 |
| 9 | EAW56981.1 | 119577385 | SH3KBP1 binding protein 1 isoform CRA\_c | 73661 | 197 | 22 |
| 10 | EAW73936.1 | 119594339 | damage-specific DNA binding protein 1 | 121371 | 172 | 14 |
| 11 | BAG59708 | 194386288 | unnamed protein product | 101054 | 172 | 10 |
| 12 | CAG33049.1 | 48145653 | PECI, (Peroxisomal 3,2-trans-enoyl-CoA isomerase; Dodecenoyl-CoA isomerase) | 39584 | 2463 | 152 |
| 13 | AAF68974.1 | 7739706 | hepatocellular carcinoma-associated antigen 64 | 21575 | 2257 | 121 |
| 14 | BAD97085.1 | 62898291 | ubiquitous mitochondrial creatine kinase precursor variant | 46935 | 2131 | 204 |
| 15 | NP\_005460.2 | 34577075 | acyl-coenzyme A thioesterase 8 | 35891 | 289 | 25 |
| 16 | AAA60561.1 | 338237 | sarcomeric mitochondrial creatine kinase precursor (EC 2.7.3.2) | 47490 | 235 | 85 |
| 17 | AAH08633 | 14250401 | actin, beta | 40978 | 232 | 18 |
| 18 | NP\_001886.1 | 4503095 | casein kinase II alpha 1 subunit isoform a | 45115 | 223 | 16 |
| 19 | NP\_004829 | 224809408 | homer protein homolog 3 isoform 1 | 39881 | 165 | 17 |
| 20 | AAH07753.1 | 14043538 | MYLK2 protein (Myosin Light Chain Kinase 2) | 27580 | 155 | 16 |
| 21 | AAH31038.1 | 21410256 | KCTD17 protein | 34767 | 391 | 17 |
| 22 | NP\_002291.1 | 4557032 | L-lactate dehydrogenase B | 36615 | 249 | 77 |
